# Mediterranean diet and depression: a population-based cohort study

**DOI:** 10.1186/s12966-021-01227-3

**Published:** 2021-11-27

**Authors:** Weiyao Yin, Marie Löf, Ruoqing Chen, Christina M. Hultman, Fang Fang, Sven Sandin

**Affiliations:** 1 Institute of Environmental Medicine, Karolinska Institutet, Box 210, 171 77 Stockholm, Sweden; 2 Department of Obstetrics and Gynecology, West China Second University Hospital, Sichuan University, Chengdu, China; 3 Department of Health, Medicine and Caring Sciences, Linköping University, Linköping, Sweden; 4 Department of Biosciences and Nutrition, Karolinska Institutet, Stockholm, Sweden; 5 School of Public Health (Shenzhen), Sun Yat-sen University, Shenzhen, China; 6 Department of Medicine Solna, Clinical Epidemiology Division, Karolinska Institutet, Stockholm, Sweden; 7 Department of Medical Epidemiology and Biostatistics, Karolinska Institutet, Stockholm, Sweden; 8 Department of Psychiatry, Ichan School of Medicine, Mount Sinai, New York, USA; 9 Seaver Autism Center for Research and Treatment at Mount Sinai, New York, USA

**Keywords:** Depression, Mental health, Mediterranean, Diet, women’s health, Cohort studies, Epidemiology

## Abstract

**Background:**

Depression imposes immense public health burden, demonstrating an urgent need of the identification of modifiable risk factors. Only a few cohort studies have analyzed the association between Mediterranean dietary pattern (MDP) and depression but with mixed results. We examined the impact of MDP on clinically ascertained depression in a large population-based dataset.

**Methods:**

In 1991/92, detailed information on diet, using a food frequency questionnaire, and potential confounding factors (body weight, height, educational attainment, smoking, previous diabetes and hypertension, and physical activity) was collected, in a random sample of 49,261 Swedish women aged 29-49. Adherence to MDP was calculated. Clinical depression was extracted from the National Patient Register. Study participants were followed up through 2012.

**Results:**

During an average follow-up of 20.4 years, 1677 incident cases of depression were diagnosed. We observed a lower risk of depression for medium (score 4-5) and high (6-9) adherence to MDP, compared with low (0-3) adherence (Medium: hazard ratio (HR) = 0.90, 95% confidence interval (CI) = 0.81-1.00; High: HR = 0.82, 95%CI = 0.71-0.94). Per unit increase of adherence, the risk of depression was reduced by 5% (HR = 0.95, 95%CI = 0.92-0.98). The association became stronger when restricting to severe form of depression (HR = 0.51, 95%CI = 0.33-0.76). The HRs were higher from age 50 onward both over the first and the second 10-year follow-up period, compared with before age 50, indicating stronger association with increasing age. Results remained after extensive sensitivity analyses.

**Conclusion:**

Higher adherence to a Mediterranean diet at middle age was associated with a lower risk of depression later in life among Swedish women.

**Supplementary Information:**

The online version contains supplementary material available at 10.1186/s12966-021-01227-3.

## Background

Worldwide, depression is one of the leading causes of disability and mortality, especially among women [1], imposing substantial impairment and immense health burden [1, 2]. The identification of modifiable risk factors for depression is therefore urgently needed. In addition to genetics [3], personality [4], and environmental factors [5], lifestyle factors such as diet have also been proposed as risk modifiers for depression [6]. Dietary factors have been indeed recommended by the International Society for Nutritional Psychiatry Research as promising modifiable targets for prevention and treatment for common mental disorders [7].

Mediterranean diet is one of the classical anti-inflammatory dietary patterns [8] and has been perceived to have benefits on multiple aspects of human health, including mental and brain health [9, 10]. This dietary pattern refers to a food profile characterized by high consumption of vegetables, fruits, legumes, nuts and complex carbohydrates, and mono-unsaturated lipids (coupled with low saturated fat consumption), moderately high consumption of fish, low consumption of dairy and meat products, and regular but low-to-moderate intake of alcohol [11]. The underlying mechanisms of action of dietary pattern on health outcomes are complex [12]. Besides inflammation, modulation of oxidative stress, mitochondrial dysfunction, the gut microbiota, tryptophan–kynurenine metabolism, the hypothalamic pituitary adrenal (HPA) axis, neurogenesis and brain-derived neurotrophic factor (BDNF) and epigenetics have also been suggested [12].

Existing observational studies have so far provided inconclusive evidence for the association between Mediterranean dietary pattern (MDP) and risk of depression [13–15]. The conflicting results may be partly explained by different study design, limited sample size, short follow-up, lack of control for potential confounders and varying definitions of depression. A meta-analysis of 16 interventional studies shows that dietary interventions (e.g., through individual and group counselling on healthy diets) is associated with improved depressive symptoms, particularly in female populations [16]. The majority of the studies included in this meta-analysis, however, used self-reported depressive symptoms. A few interventional studies of clinically diagnosed depression indicate larger beneficial effects of dietary interventions among people with higher baseline level of depression [17–19]. Although previous studies were mostly performed in western countries, no research has yet been carried out in Nordic populations.

The aim of this study was to examine the association of adherence to MDP with the risk of depression in a large population-based cohort in Sweden with detailed information about confounding factors and clinically ascertained depression diagnosis.

## Materials and methods

Between August 1991 and March 1992, a random sample of 96,000 women at age 29-49 and residing in the Uppsala healthcare region of Sweden was drawn from the Total Population Register by Statistics Sweden. These women were invited to participate in the Women’s Lifestyle and Health (WLH) study, through answering a comprehensive questionnaire including food frequency questionnaire (FFQ) at the cohort entry [20]. Among the women invited, 49,261 returned the questionnaire and were enrolled in the study. All eligible participants were followed from cohort entry as defined by return of the questionnaire in 1991/92 until the first diagnosis of depression, date of emigration, death, or December 31, 2012, whichever came first, through cross linkages to the Swedish Patient Register (for ascertainment of depression) and Total Population Register (for ascertainment of emigration and death), using the individually unique personal identity numbers [21].

### Adherence to MDP

At cohort entry, the study participants were asked to recall their dietary habits during the six months before enrolment, through answering an FFQ, which assessed the frequency and quantity of consumption of approximately 80 food items and beverages [22]. The consumption (grams/day) of each item and total energy intake (kilo-Joule (kJ)/day) were calculated using the Swedish National Food Administration database [23]. To measure adherence to MDP, we used the scale proposed by Trichopoulou et al. [9, 24] which has been used extensively in different studies including the WLH study [25].

Altogether, nine food groups were constructed as index dietary components, namely vegetables, fruits and nuts, cereals, legumes, dairy products, fish and seafood, meat, alcohol, and monounsaturated-to-saturated fat (M/S) ratio. For dietary components that are presumed to be beneficial (i.e., vegetables, fruits and nuts, cereals, legumes, fish and seafood, and a high M/S ratio), we scored a woman that consumed below the median level of the entire cohort as “0” and a woman that consumed at or above the cohort median as 1. For dietary components that are presumed to be less beneficial (i.e., dairy and meat products), a consumption level below the cohort median was given a score of 1 whereas a consumption level at or above the cohort median was given a score of 0. A moderate level of alcohol consumption (5-25 g/day) was scored 1, or 0 otherwise. Scores on all nine components were then summed up as a proxy for adherence to MDP, with the value 0 as the minimal and 9 as the maximal adherence [24].

### Diagnosis of depression

The outcome of the study was the first clinical diagnosis of depression during follow-up. The Swedish Patient Register includes nationwide complete information on inpatient psychiatric care since 1973 and outpatient specialist care since 2001, updated on a daily basis [26]. A clinical diagnosis of depression was identified using the Swedish revisions of the International Classification of Diseases codes (ICD-7: 301.1, ICD-8: 296.2, ICD-9: 296B, and ICD-10: F32 and F33). The date of first hospital visit concerning depression was used as date of diagnosis for depression.

In a sensitivity analysis, we used both a broader (either ≥1 dispense of selective serotonin reuptake inhibitors (SSRIs) or clinical diagnosis of depression) and a narrower (both dispense of SSRIs and clinical diagnosis of depression) definition to assess the soundness of the main results. Information on dispense of SSRIs was derived from the Swedish Prescribed Drug Register (nationwide available since July 2005) using the Anatomical Therapeutic Chemical classification code N06AB. We also used ICD-10 codes F32.2, F32.3, F33.2 and F33.3 to identify severe depression, to assess if the role of adherence to MDP would differ for severe depression.

### Covariates

We considered a range of demographic factors, lifestyle factors, anthropometric profile, and medical history as potential confounders of the studied association, including age (years, continuous), calendar year of birth (continuous), body weight (kg, continuous), height (cm, continuous), total years of education (years, continuous), smoking status (never, former or current), previous diabetes and hypertension (yes or no), as well as level of physical activity (on a 5-point scale ranging from mainly sitting as level 1 to vigorous physical activity as level 5), all collected from the questionnaires at baseline.

### Statistical analysis

We calculated incidence rates of depression standardized by age using all person-time experienced by the entire cohort as the standard. Association of adherence to MDP with the risk of depression was estimated by hazard ratios (HRs) and 95% profile likelihood confidence intervals (CIs) obtained from the Cox models. The underlying time scale was attained age [27]. Adherence to MDP was analyzed both as a categorical (0-3 low, 4-5 medium, or 6-9 high) and continuous (0-9) variable. In the minimally adjusted model, we adjusted for year of birth (1942-46, 1947-51, 1952-56, or 1957-62). In the fully adjusted model, we additionally adjusted for body mass index (BMI, < 25, 25-29.9, or ≥ 30 kg/m^2^ calculated from weight and height) [28], years of education (0-10, 11-13, or > 13), physical activity (very low, low, moderate, high, or very high), smoking (never, former, or current), diabetes history (yes or no), hypertension history (yes or no), and total energy intake as a continuous variable (kJ/day). Since effect of diet can expect to increase cumulatively with higher age, we repeated the analyses in women younger than 50 and in women aged 50 and older. The cumulative incidence rate of depression over age by adherence to MDP was plotted using Kaplan-Meier method. Natural cubic splines were fitted to display the trend of depression risk across MDP score (0-9), adjusted for attained age, birth year, BMI, smoking, physical activity, education, diabetes, hypertension, and total energy intake.

### Sensitivity and supplementary analyses

We tested the robustness of our results through a series of sensitivity analyses. To rule out the possibility of reverse causation, we excluded the first two or five years of follow-up. To address the concern for residual confounding due to a single assessment of dietary habit, we limited the follow-up time to the end of 2002 to estimate the association over the first 10 years of follow-up. We further repeated the age-specific analysis within the first 10 years and the second 10 years of follow-up respectively. We used alternative definitions for depression (broader or narrower definition, and severe depression). To address the influence of other psychiatric comorbidity, we first adjusted the analysis for history of any other psychiatric disorders (ICD-7: 300-326, ICD-8: 290-315, ICD-9: 290-319 and ICD-10: F10-99, excluding ICD codes for depression) before the end of follow-up, and then performed another analysis restricted to women without any psychiatric history before enrolment. The correlations between the nine dietary components and adherence to MDP score were calculated using Spearman’s rank correlation coefficients. To check the influence of different food components, we performed another analysis by excluding the nine components one by one from the MDP score. Finally, given the potential distinct health effects of red and white meat, we re-calculated the adherence score based only on red and processed meat [29] instead of all kinds of meat products, and also separately assessed the association of red meat with risk of depression.

The assumption of proportional hazards was assessed by examining the standardized Schoenfeld residuals [30]. All statistical tests were performed on the two-sided 5% level of significance, corresponding to a two-sided 95% CI. We did not perform any adjustment of *p*-values for multiplicity of statistical tests. Data management was performed using SAS software version 9. 4 (SAS institute Inc., Cary, NC, USA). Survival analyses were performed using SAS software version 9.4. The cumulative incidence rate and the age-specific analysis were performed using STATA version 14 (StataCorp LP, College Station, TX, USA). SAS codes for the Cox regression analyses are presented in the online appendix. The present study was approved by Regional Ethical Review Board in Stockholm, Sweden.

## Results

### Baseline characteristics

Of the 49,261 women that returned questionnaire, we excluded 1049 women who emigrated out of Sweden before enrollment, 567 women who did not answer the FFQ, 100 women with prevalent clinically confirmed depression at enrolment, and 603 women with total energy intake below the 1st (1847 kJ/day) or above the 99th (12,474 kJ/day) percentiles of the cohort. The final study cohort comprised of 46,942 women. After exclusion of women with missing data on any of the covariates, 42,515 women with a mean age of 39.5 years (standard deviation = 5.6) remained in the final analysis (Fig. S1). No major differences existed between the women excluded due to missing data and the women included in the final analysis (Table S1).

During the average follow-up of 20.4 years, we identified 1677 women with incident depression, leading to an incidence rate of 1.94 per 1000 person-years. Table 1 shows the baseline characteristics of the study participants according to the three categories of adherence to MDP score. Women with a high adherence tended to be older, had higher educational attainment, more physically active, non-smoking, and with a higher total energy intake, compared with women with a low adherence.

**Table 1:** Baseline characteristics of the study women ^a^ by adherence to the Mediterranean dietary pattern

	Adherence to Mediterranean Dietary Pattern, N (%)		
	Low (0-3)	Medium (4-5)	High (6-9)
**Age at enrolment** (years)			
29-34	4032 (27.9)	4336 (23.4)	1825 (19.2)
35-39	3830 (26.5)	4828 (26.0)	2291 (24.1)
40-44	3529 (24.4)	4963 (26.8)	2710 (28.5)
45-49	3062 (21.2)	4413 (23.8)	2696 (28.3)
**Body mass index** (kg/m^2^)			
< 25	10,456 (72.3)	13,258 (71.5)	6997 (73.5)
25-30	3103 (21.5)	4182 (22.6)	2080 (21.8)
≥ 30	894 (6.2)	1100 (5.9)	445 (4.7)
**Years of education**			
0-10	4942 (34.2)	5347 (28.8)	2439 (25.6)
11-13	5857 (40.5)	7201 (38.8)	3486 (36.6)
> 13	3654 (25.3)	5992 (32.3)	3597 (37.8)
**Smoking**			
Never	5563 (38.5)	7895 (42.6)	4114 (43.2)
Former	3948 (27.3)	5465 (29.5)	3145 (33.0)
Current	4942 (34.2)	5180 (27.9)	2263 (23.8)
**Physical activity**			
Very low	812 (5.6)	693 (3.7)	257 (2.7)
Low	1732 (12.0)	1961 (10.6)	853 (9.0)
Moderate	8852 (61.2)	11,082 (59.8)	5425 (57.0)
High	2076 (14.4)	3219 (17.4)	1963 (20.6)
Very high	981 (6.8)	1585 (8.5)	1024 (10.8)
**Diabetes**			
No	14,267 (98.7)	18,299 (98.7)	9391 (98.6)
Yes	186 (1.3)	241 (1.3)	131 (1.4)
**Hypertension**			
No	13,139 (90.9)	16,799 (90.6)	8641 (90.7)
Yes	1314 (9.1)	1741 (9.4)	881 (9.3)
**Total energy intake** (kJ/day) ^b^	6160 (1850)	6610 (1880)	6940 (1770)

^a^ There were 49,261 participants who returned the questionnaire in 1991-1992. Among them, 2319 women were excluded before cohort entry, leaving 46,942 in the cohort. After further exclusion of 4427 women with missing values in the covariates, 42,515 women were included in final analysis

^b^ Mean (standard deviation)

### Adherence to MDP and risk of depression

Compared with low adherence to MDP (score 0-3), there was a statistically significantly decreased risk of depression for both medium (score 4-5) (HR_adj_ = 0.90, 95%CI = 0.81-1.0) and high (score 6-9) (HR_adj_ = 0.82, 95%CI = 0.71-0.94) adherence to MDP in all models (Table 2). Per unit increase of adherence to MDP, there was a 5% lower risk of depression in the fully adjusted models. The observed protective effect appeared to increase with age. The HRs were higher from age 50 onward (HR_adj_ = 0.76, 95%CI = 0.64-0.89, high vs low adherence), compared with before age 50 (HR_adj_ = 1.00, 95%CI = 0.78-1.27, high vs low adherence) (Table 2).

**Table 2:** The association between adherence to the Mediterranean dietary pattern and the risk of depressive disorder

	Depression		
Adherence to MDP	Cases/participants, N	Minimally adjusted HR (95% CI) ^a^	Fully adjusted HR (95% CI) ^b^
Low (0-3)	646/14,453	Reference category	Reference category
Medium (4-5)	710/18,540	0.86 (0.77-0.96)	0.90 (0.81-1.0)
High (6-9)	321/9522	0.77 (0.67-0.88)	0.82 (0.71-0.94)
Per unit increase	1677/42,515	0.94 (0.91-0.97)	0.95 (0.92-0.98)
**Subset: Women aged < 50 at follow-up:**			
Low (0-3)	223	reference category	reference category
Medium (4-5)	221	0.87 (0.72-1.05)	0.94 (0.77-1.13)
High (6-9)	99	0.88 (0.70-1.12)	1.00 (0.78-1.27)
**Subset: Women aged ≥ 50 at follow-up:**			
Low (0-3)	423	reference category	reference category
Medium (4-5)	489	0.85 (0.75-0.97)	0.88 (0.77-1.00)
High (6-9)	222	0.72 (0.61-0.85)	0.76 (0.64-0.89)

^a^ HRs (hazard ratios) and 95% CI (confidence intervals) were derived from Cox models using attained age as the time scale, adjusted for year of birth (1942-46, 1947-51, 1952-56 and 1957-62).

^b^ HRs (hazard ratios) and 95% CI (confidence intervals) were derived from Cox models using attained age as the time scale, adjusted for year of birth, body mass index, smoking, physical activity, total energy intake, years of education, history of diabetes and hypertension.

*MDP* Mediterranean dietary pattern

**Fig. 1:**
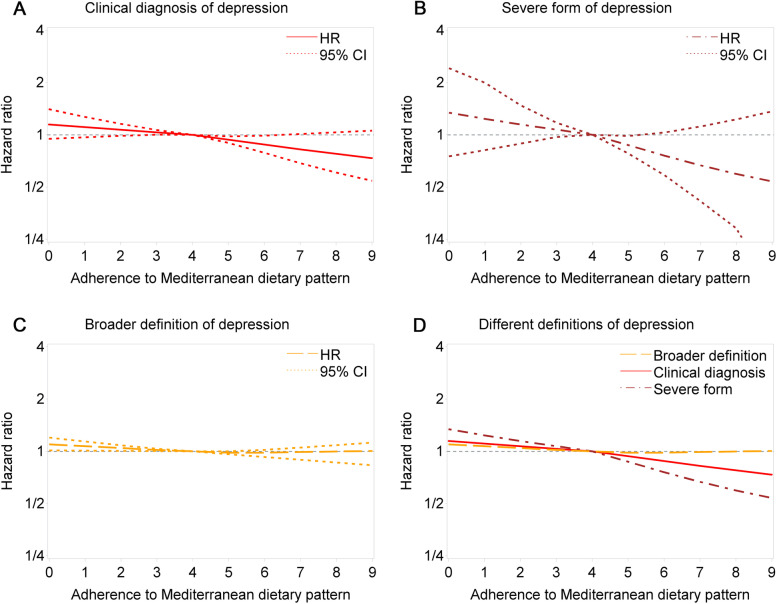
“Hazard ratios of depression by adherence to Mediterranean dietary pattern score (0-9) based on different definitions of depression”. Relative risk of depression (point estimates with pointwise two-sided 95% confidence intervals), by adherence to Mediterranean dietary pattern score (0-9), referenced on a score of 4. **A** Clinical diagnosis of depression; **B** Severe form of depression; **C** Broader definition of depression (at least one dispense of SSRIs or clinical diagnosis of depression); **D** Combined splines in the Fig. 1A-C. Estimates were derived from natural cubic splines, adjusted for attained age, birth year, body mass index, smoking, physical activity, total energy intake, years of education, and history of diabetes and hypertension. Adherence to Mediterranean dietary pattern score was calculated on a 9-point scale ranging from 0 as the minimal to 9 as the maximal adherence

**Fig. 2:**
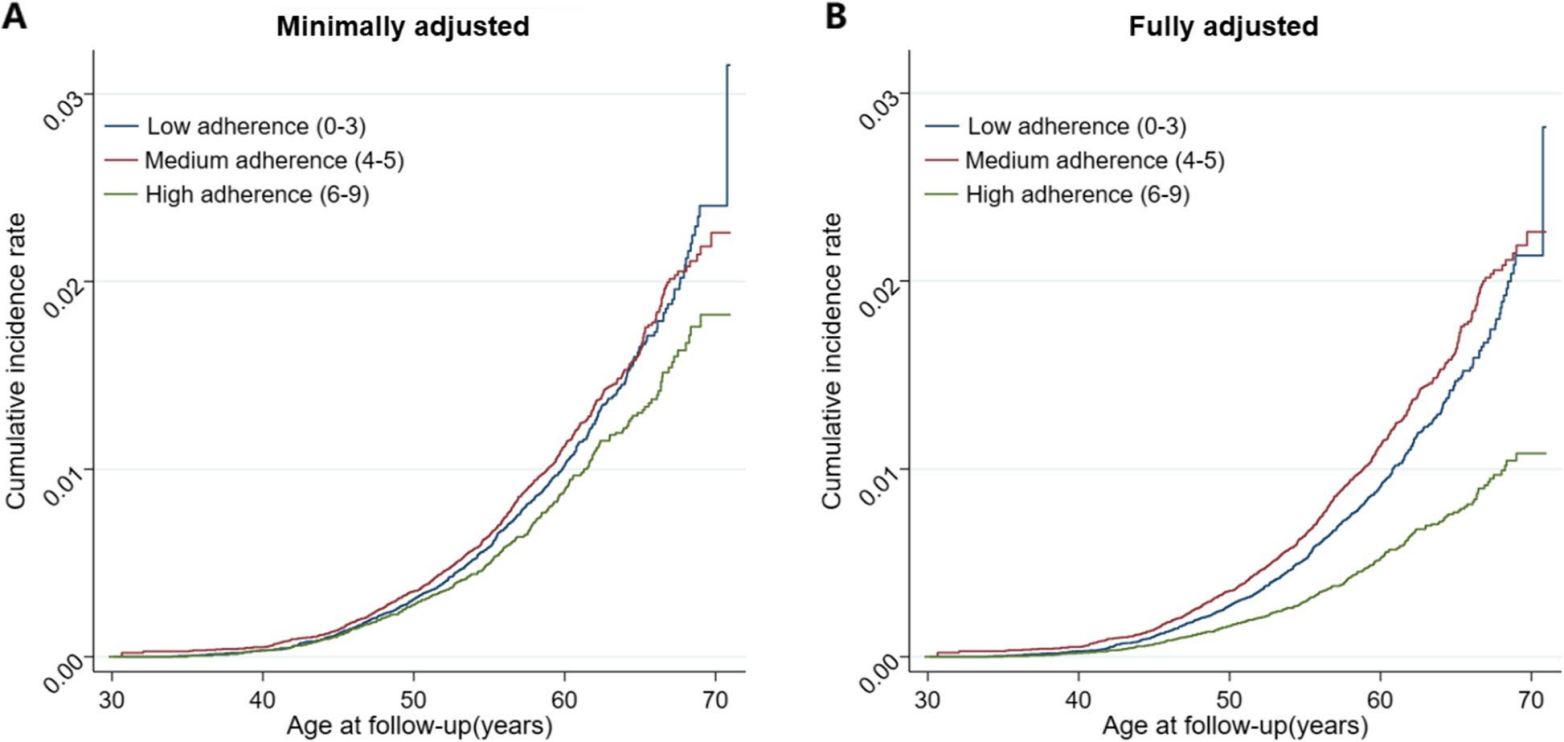
“Kaplan-Meier curves of depression by adherence to Mediterranean dietary pattern”. Cumulative incidence rate of depression with age of follow-up (years) by adherence to Mediterranean dietary pattern using Kaplan-Meier method. **A** Minimally adjusted Cox model, adjusted for attained age and birth year; **B** Fully adjusted Cox model, further adjusted for body mass index, smoking, physical activity, total energy intake, years of education, and history of diabetes and hypertension

The fully adjusted spline regression showed a similar result pattern (Fig. 1). Examination of the age-specific cumulative incidence rate of depression indicated stronger associations with increasing age and increasing adherence to MDP (Fig. 2).

### Sensitivity and supplementary analyses

Excluding the first two or five years of follow-up provided essentially similar results (Table S2). Compared with the main model, the association appeared to be weaker when using the broader definition of depression (fully-adjusted HR = 0.94, 95%CI = 0.88-1.00, high vs low adherence), but stronger when using the narrower definition of depression (fully-adjusted HR = 0.83, 95%CI = 0.70-0.97, high vs low adherence) (Table 3). The strongest association was noted for severe depression (fully-adjusted HR = 0.51, 95%CI = 0.33-0.76, high vs low adherence). Further adjustment for psychiatric comorbidity (Table S3) or restricting analysis to women without any psychiatric disorders before enrolment (Table S4) did not change the results either. Excluding other kinds of meat (e.g., white meat) from the adherence score led to largely similar results (Table S5). The correlations between individual dietary components and adherence to MDP are shown in Table S6. The results remained robust after exclusion of individual food components from the adherence score (Fig. S2). There was no strong support for lack of proportional hazards (Fig. S3). The pattern of stronger association with age was consistent over the first 10 years and the second 10 years of follow-up (Table S7).

**Table 3:** The association between adherence to the Mediterranean dietary pattern and risk of depression based on different definitions

Adherence to MDP	Broad definition ^a^			Narrow definition ^b^			Severe depression ^c^		
	Case/participants, N	Minimally adjusted HR ^d^	Fully adjusted HR ^e^	Case, N	Minimally adjusted HR	Fully adjusted HR	Case, N	Minimally adjusted HR	Fully adjusted HR
Low(0-3)	2644/14,453	ref.	ref.	465	ref.	ref.	97	ref.	ref.
Medium(4-5)	3134/18,540	0.91 (0.86-0.96)	0.94 (0.89-0.99)	499	0.84 (0.74-0.95)	0.88 (0.78-1.01)	97	0.79 (0.60-1.05)	0.79 (0.60-1.06)
High(6-9)	1568/9522	0.89 (0.83-0.95)	0.94 (0.88-1.00)	227	0.76 (0.64-0.89)	0.83 (0.70-0.97)	31	0.51 (0.34-0.76)	0.51 (0.33-0.76)
Per unit increase	7346/42,515	0.97 (0.96-0.98)	0.99 (0.97-1.00)	1191	0.93 (0.89-0.96)	0.95 (0.91-0.98)	225	0.89 (0.82-0.97)	0.89 (0.82-0.97)

^a^ Broad definition: ≥1 dispense of SSRIs or clinical diagnosis of depression

^b^ Narrow definition: Both dispense of SSRIs and clinical diagnosis of depression

^c^ Severe depression: Based on International Classification of Disease codes: ICD-10 F32.2, F32.3, F33.2 and F33.3

^d^ HRs (hazard ratios) and 95% CI (confidence intervals) were derived from Cox models using attained age as the time scale, adjusted for year of birth (1942-46, 1947-51, 1952-56 and 1957-62)

^e^ HRs (hazard ratios) and 95% CI (confidence intervals) were derived from Cox models using attained age as the time scale, adjusted for year of birth, body mass index, smoking, physical activity, total energy intake, years of education years, and history of diabetes and hypertension

*MDP* Mediterranean dietary pattern

## Discussion

In this, to date, largest prospective cohort study examining the association between adherence to Mediterranean-style diet and risk of clinically ascertained depression, we observed a reduction in the risk of depression in relation to higher adherence to Mediterranean diet, compared with a lower adherence, in a dose-response pattern. Among younger women, whose causes of depression are mainly depression with probable genetic influences [31, 32], there was no, or only a small, association of the Mediterranean diet score with depression. The results remained robust after a detailed adjustment for potential confounding factors and after an extensive set of sensitivity analyses. The highest reduction in risk was observed for severe depression.

There has been a rapid growth in research concerning the role of diet in depression in recent years [6], centered on its modulation of inflammatory level [33]. As a supportive evidence, the US Nurses’ Health Study observed a positive association between increasing adherence to inflammatory diet and risk of depression [34]. Conversely, Mediterranean diet has been frequently referred as a typical food pattern with a lower level of inflammation [8], and was found to be inversely associated with risk of depression in our study. Other hypotheses are also proposed as explanations, including pathways in the oxidative and antioxidant defense systems, brain plasticity, microbiota-gut-brain axis, mitochondrial dysfunction, tryptophan–kynurenine metabolism, neurogenesis and BDNF, and epigenetics [12]. The action by diet affecting mental health is likely multifaceted and interacting, not restricted to only one pathway [12]. However, the majority of identified mechanisms nowadays are derived from animal studies; data of clinically ascertained depression in human beings are lacking.

Previous cohort studies among women showed mixed results on the association between MDP and risk of depression [35–38]. Unlike most earlier studies, which used symptom scales or measures of self-reported depression, our study used clinically ascertained depression. Moreover, the large sample size of the present study allowed us to perform detailed adjustment for potential confounding factors and several sensitivity analyses to challenge assumptions used in the analysis, including the influence from different definitions of depression. When the broader depression definition was used (≥1 dispense of SSRIs or clinical diagnosis of depression), the protective effect from MDP diminished, whereas results remained essentially unchanged with the narrower definition (both dispense of SSRIs and clinical diagnosis of depression), compared with the main analysis. The effect became even more evident when we included only the most severe depression. Similar pattern was described in the Nurses’ Health Study between inflammatory diet and depression, with the stronger effect noted for the narrow definition whereas relatively weaker effect noted for the broad definition of depression [34]. Furthermore, the age-specific analysis showed a stronger effect with increasing age, indicating a potentially accumulative benefit of MDP. This might partly explain the null association in some of the previous studies with a shorter follow-up period [38]. Finally, our finding of reduced risk of clinical depression with MDP is consistent with recent interventional studies which exhibited reduced depressive symptoms after dietary interventions, especially for those of more severe level of depression [16–19].

In our cohort, women with a higher adherence to Mediterranean diet were more likely to have healthier lifestyle behaviors, characterized by being more physically active, of higher educational level, and with lower prevalence of smoking. Our extensive database allowed us to further adjust for these lifestyle factors which slightly attenuated the beneficial effect from MDP. Similarly, metabolic diseases can result in dietary habits which could modulate the risk of depression in turn [7, 14]. As obesity is known to stimulate chronic pro-inflammatory status, it is a plausible pathway linking together diet and risk of depression [39]. In our study, the results remained robust after adjusting for BMI.

Strengths of our study include the population-based sample, large cohort size, prospective ascertainment of clinically defined depression and long and virtually complete follow-up. Risk of selection bias was limited by the use of data from a health care system with equal access and recorded information on all cohort participants, independent from sociodemographic background. The same system allowed us to screen for presence of psychiatric disorders before the cohort entry.

Our study also has limitations. When at an early stage of depression, women might be more likely to have an unhealthy dietary pattern, indicating a possibility of reverse causation. To rule out potential impact of subclinical depression, we performed lag-time analyses and found that the association remained essentially unchanged after excluding the first two or five years of follow-up. Additionally, we adjusted for history of other psychiatric disorders prior to depression diagnosis in one analysis and excluded women with any psychiatric history before enrolment in another sensitive analysis. Both analyses showed largely comparable results. In the main analysis, we used calendar year of birth, BMI and years of education as categorical variables. In another model adjusting for these variables as continuous variables, we obtained very similar results as the ones from the original analyses (data not shown). The dietary pattern was measured only at enrolment. The long lag between exposure and outcome is therefore a concern because dietary pattern of the participants might have changed during the follow-up. As a result, there is a chance of misclassification of the exposure. Nevertheless, researchers have noticed that individual dietary habit is usually maintained lifelong and unlikely to change greatly over time [40, 41]. To compare the risk pattern over the first 10 years and the second 10 years of follow-up, we repeated the age-specific analysis within the two 10-year follow-up periods and found consistently a lower risk of depression in relation to greater adherence to MDP. The magnitude of the association increased with increasing age, potentially suggesting a cumulative protective effect from MDP on depression across life span. Given the observational nature of the study, residual confounding due to unknown or unmeasured confounders such as stress-related factors may still have some influence on our results.

Diet, which every one of the population is exposed to, has been shown associated with overall health and various specific diseases, e.g. cardiovascular disease, cancer, premature mortality and, in this study, depression [42]. Considering the health burden of depression worldwide, these results are of great public health importance. More researchers have emphasized the importance of dietary pattern, instead of isolated nutrients [43], to achieve interactional effects and to convey easy-to-understand information to the public [44]. This is in line with the intention of this study which is to raise awareness of the importance of healthy dietary pattern on mental health. Although MDP is prevalent in Mediterranean countries, the traditional dietary habits in the Scandinavian population are however not a typical Mediterranean style. Compared with a Greek cohort (EPIC) [45], the median intake levels of vegetables and fruits and nuts were much lower in our cohort (*Table S6*). Due to the relatively low absolute prevalence of MDP, the adherence to a dietary pattern that approximates MDP might bring about even greater benefits to the health of a population outside the Mediterranean region, like Sweden.

## Conclusion

Higher adherence to a Mediterranean diet at middle age was associated with a lower risk of depression later in life among Swedish women.

## Supplementary Information

**Additional file 1 Fig. S1** The Women’s Lifestyle and Health Cohort. **Fig. S2** The robustness of the adherence score of Mediterranean dietary pattern. **Fig. S3** Test of assumption of proportional hazards by the standardized Schoenfeld residuals. **Table S1** Baseline characteristics of women excluded due to missing value on main covariates by adherence to the Mediterranean dietary pattern. **Table S2** The association between adherence to the Mediterranean dietary pattern and the risk of depression (excluding the first 2 or 5 years of follow-up). **Table S3** The association between adherence to the Mediterranean dietary pattern and the risk of depression adjusted for history of other psychiatric disorder. **Table S4** The association between adherence to the Mediterranean dietary pattern and the risk of depression among women without psychiatric history. **Table S5** The association between adherence to the Mediterranean dietary pattern (based on red and processed meat) and the risk of depression. **Table S6** The consumption of different components and their correlations with the score of Mediterranean dietary pattern. **Table S7** The age specific analysis over the first 10 years and the second 10 years of follow-up.

## Data Availability

Data described in the manuscript, code book, and analytic code will be made available upon request pending. SAS codes for the main analyses are presented in the online appendix.

## Abbreviations

MDP: Mediterranean dietary pattern
SSRIs: selective serotonin reuptake inhibitors
WLH: women’s lifestyle and health
HR: hazard ratio
CI: confidence interval
ICD: International Classification of Diseases
