# Supplementary material for: Mediterranean diet and depression: a population-based cohort study
Source: Int J Behav Nutr Phys Act. 2021 Nov 27;18:153. doi: 10.1186/s12966-021-01227-3 (PMC8627099; doi:10.1186/s12966-021-01227-3)
Supplement: Supplementary file 1 — Additional file 1 Fig. S1 The Women’s Lifestyle and Health Cohort. Fig. S2 The robustness of the adherence score of Mediterranean dietary pattern. Fig. S3 Test of assumption of proportional hazards by the standardized Schoenfeld residuals. Table S1 Baseline characteristics of women excluded due to missing value on main covariates by adherence to the Mediterranean dietary pattern. Table S2 The association between adherence to the Mediterranean dietary pattern and the risk of depression (excluding the first 2 or 5 years of follow-up). Table S3 The association between adherence to the Mediterranean dietary pattern and the risk of depression adjusted for history of other psychiatric disorder. Table S4 The association between adherence to the Mediterranean dietary pattern and the risk of depression among women without psychiatric history. Table S5 The association between adherence to the Mediterranean dietary pattern (based on red and processed meat) and the risk of depression. Table S6 The consumption of different components and their correlations with the score of Mediterranean dietary pattern. Table S7 The age specific analysis over the first 10 years and the second 10 years of follow-up. [file 12966_2021_1227_MOESM1_ESM.docx]

# **Supplementary Appendix**

**CONTENTS**

**Figures**

**Figure S1** The Women’s Lifestyle and Health Cohort

**Figure S2** The robustness of the adherence score of Mediterranean dietary pattern

**Figure S3** Test of assumption of proportional hazards by the standardized Schoenfeld residuals

**Tables**

**Table S1** Baseline characteristics of women excluded due to missing value on main covariates by adherence to the Mediterranean dietary pattern

**Table S2** The association between adherence to the Mediterranean dietary pattern and the risk of depression (excluding the first 2 or 5 years of follow-up)

**Table S3** The association between adherence to the Mediterranean dietary pattern and the risk of depression adjusted for history of other psychiatric disorder

**Table S4** The association between adherence to the Mediterranean dietary pattern and the risk of depression among women without psychiatric history

**Table S5** The association between adherence to the Mediterranean dietary pattern (based on red and processed meat) and the risk of depression.

**Table S6** The consumption of different components and their correlations with the score of Mediterranean dietary pattern.

**Table S7** The age specific analysis over the first 10 years and the second 10 years of follow-up

**Codes**

SAS codes for the main Cox regression analyses.

**Supplementary Figures**


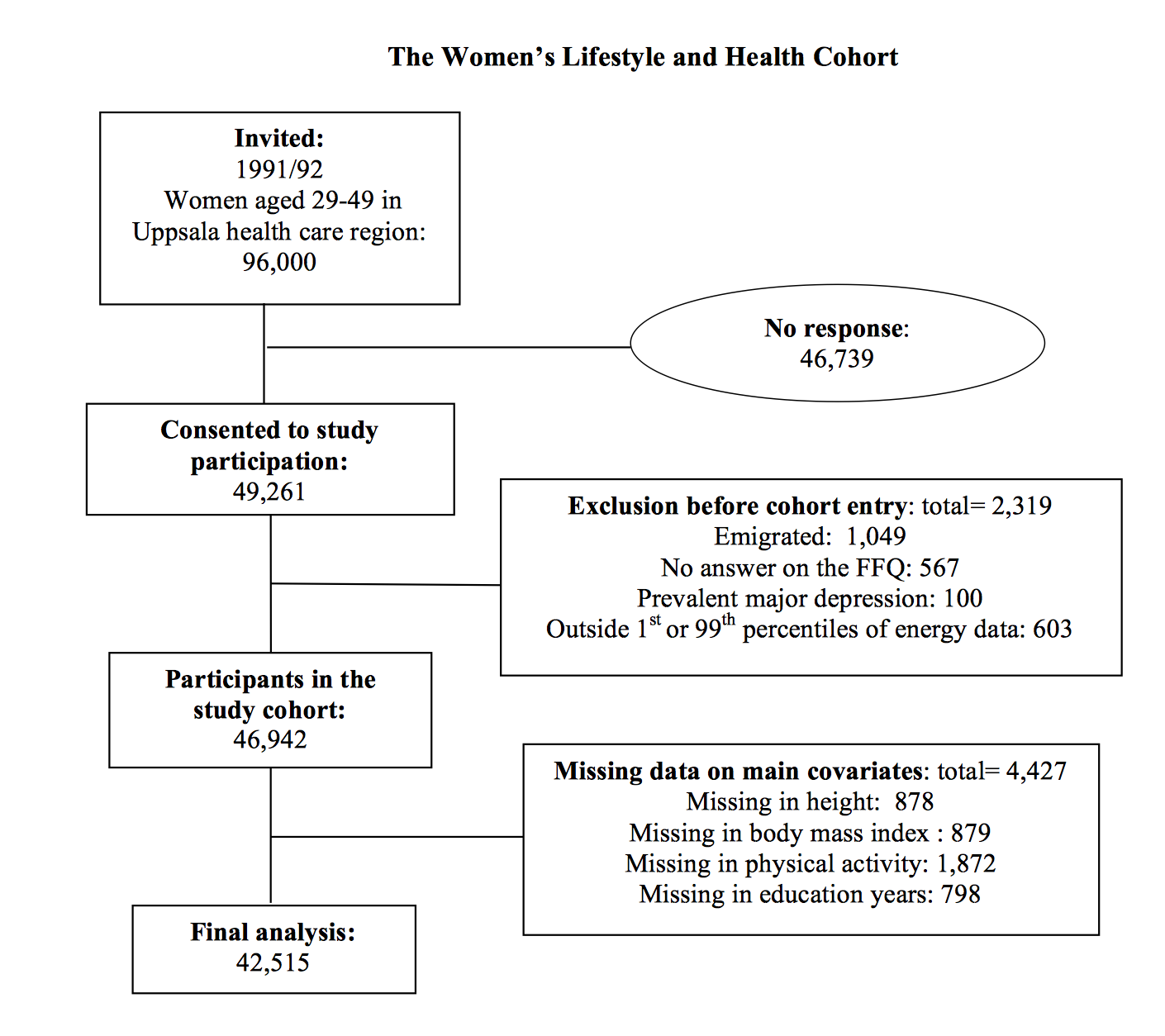


**Figure S1**: Flow chart of recruitment and exclusion of the study cohort. In 1991/92, a random sample of 96,000 women aged 29-49 and residing in the Uppsala healthcare region were invited to participate in the study through answering a comprehensive questionnaire distributed by post. Among the women invited, 49,261 returned the questionnaire and were enrolled in the study. With completion of new linkage in December 2012, the final analysis included 42,515 participants, after excluding women emigrated before cohort entry, with no answer on the FFQ, with prevalent depression, with extreme energy intakes and with missing data on any of the model covariates. FFQ: Food frequency questionnaire.

**
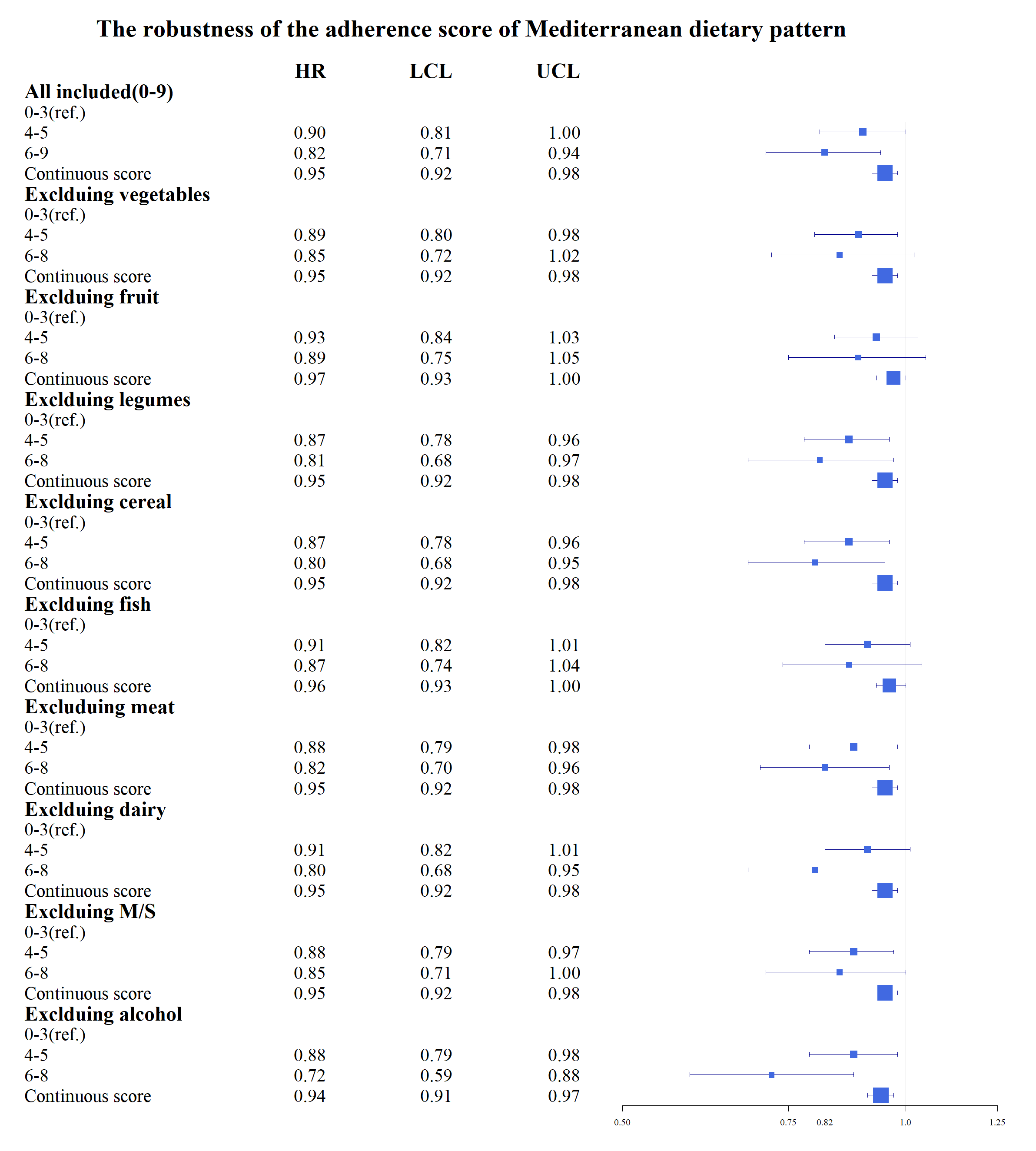
**

**Figure S2**  To check the influence of different food components by excluding the nine components one by one from the MDP score. HRs (hazard ratios) and 95% confidence intervals (LCL: lower confidence interval; UCL: upper confidence interval) were derived from Cox models using attained age as the time scale, adjusted for year of birth, body mass index, smoking habit, physical activity, total energy intake, education years, diabetes and hypertension.


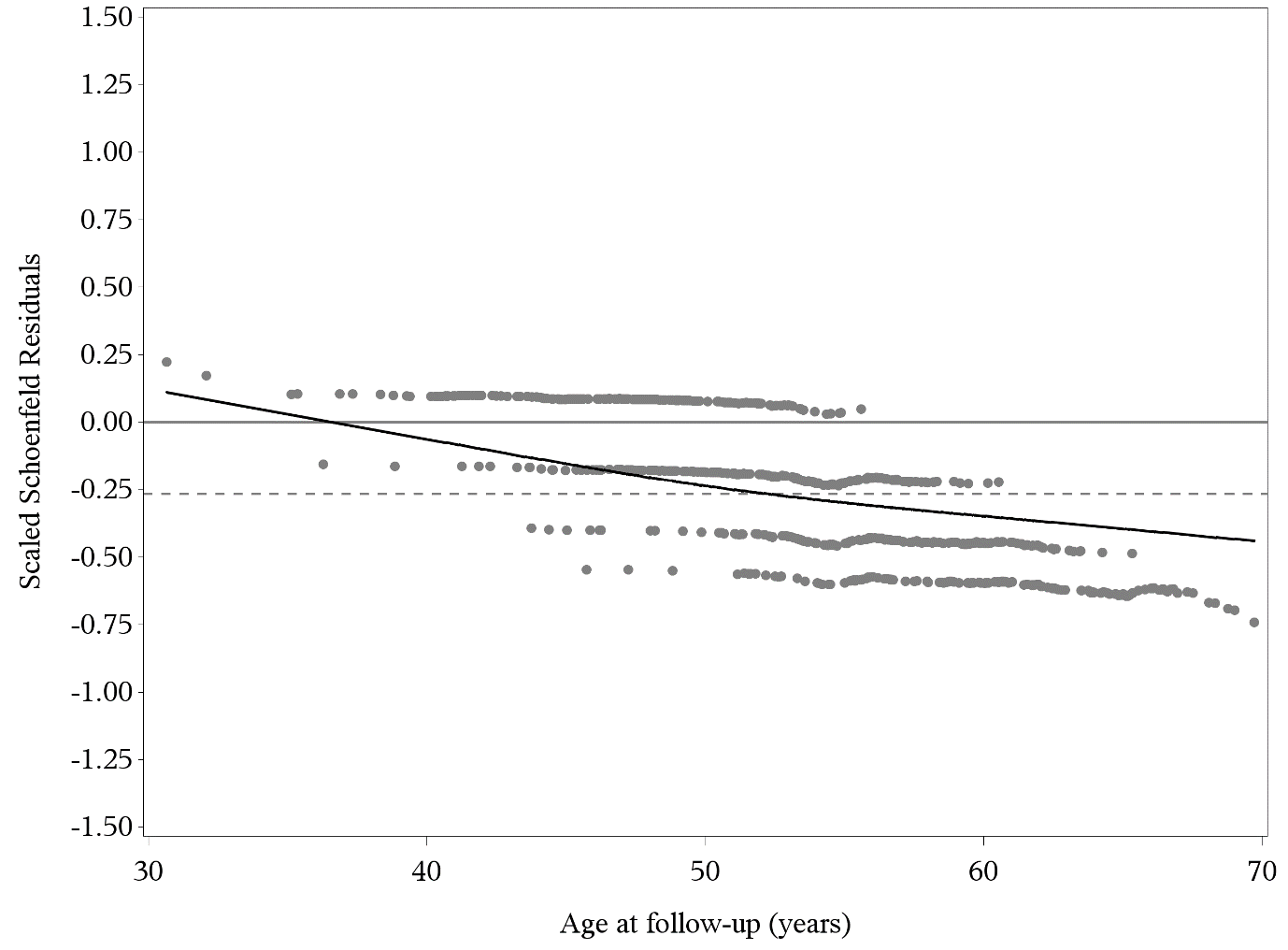
**Test of assumption of proportional hazards**

**Figure S3**: Test of assumption of proportional hazards, comparing high adherence (6-9) to low adherence (0-3) to the Mediterranean dietary pattern, examined by the standardized Schoenfeld residuals.

**Supplementary Tables**

**Table S1. Baseline characteristics of women excluded due to missing value on main covariates by adherence to the Mediterranean dietary pattern^1^**

|  | Adherence to Mediterranean Dietary Pattern, N (%)^2^ | | |
| --- | --- | --- | --- |
|  | Low (0-3) | 4-5 | Low (0-3) |
| **Age at enrolment** (years) |  |  |  |
| 29-34 | 910 (54.9) | 903 (48.5) | 413 (45.5) |
| 35-39 | 235 (14.2) | 307 (16.5) | 139 (15.3) |
| 40-44 | 261 (15.7) | 306 (16.4) | 166 (18.3) |
| 45-49 | 252 (15.2) | 345 (18.5) | 190 (20.9) |
| **Body mass index** (kg/m^2^) |  |  |  |
| <25 | 790 (47.6) | 824 (44.3) | 408 (44.9) |
| 25-30 | 184 (11.1) | 202 (10.9) | 112 (12.3) |
| =>30 | 59 (3.6) | 62 (3.3) | 29 (3.2) |
| Missing value | 625 (37.7) | 773 (41.5) | 359 (39.5) |
| **Years of education** |  |  |  |
| 0-10yrs | 447 (27.0) | 408 (21.9) | 168 (18.5) |
| 11-13yrs | 618 (37.3) | 629 (33.8) | 296 (32.6) |
| >13yrs | 292 (17.6) | 439 (23.6) | 215 (23.7) |
| Missing value | 301 (18.2) | 385 (20.7) | 229 (25.2) |
| **Smoking** |  |  |  |
| Never | 670 (40.4) | 823 (44.2) | 428 (47.1) |
| Former | 484 (29.2) | 572 (30.7) | 291 (32.0) |
| Current | 504 (30.4) | 466 (25.0) | 189 (20.8) |
| **Physical activity** |  |  |  |
| Very low | 72 (4.3) | 66 (3.5) | 30 (3.3) |
| low | 95 (5.7) | 101 (5.4) | 43 (4.7) |
| Moderate | 515 (31.1) | 635 (34.1) | 315 (34.7) |
| high | 89 (5.4) | 162 (8.7) | 88 (9.7) |
| Very high | 42 (2.5) | 72 (3.9) | 55 (6.1) |
| Missing value | 845 (51.0) | 825 (44.3) | 377 (41.5) |
| **Diabetes** |  |  |  |
| No | 1,630 (98.3) | 1,838 (98.8) | 899 (99.0) |
| Yes | 28 (1.7) | 23 (1.2) | 9 (1.0) |
| **Hypertension** |  |  |  |
| No | 1,503 (90.7) | 1,680 (90.3) | 821 (90.4) |
| Yes | 155 (9.3) | 181 (9.7) | 87 (9.6) |
| **Total energy intake**  Mean (SD), KJ/day | 6,040 (1,910) | 6,560 (2,020) | 6,990 (1,840) |

^1^4,427 participants were excluded due to missing value on the model covariates.

SD: Standard deviation, KJ: Kilo Joule, N: Number of women; kg: Kilo, m2: Square mete

**Table S2. The association between adherence to the Mediterranean dietary pattern and the risk of depression (excluding the first 2 or 5 years of follow-up)**

|  | Depression | | | | | |
| --- | --- | --- | --- | --- | --- | --- |
| Adherence to MDP | Excluding the first 2 years of follow-up | | | Excluding the first 5 years of follow-up | | |
|  | Case/participants | Minimally adjusted HR (95% CI)^1^ | Fully adjusted HR (95%)^2^ | Case/participants | Minimally adjusted HR (95% CI) | Fully adjusted HR (95%) |
| Low (0-3) | 644/14398 | Reference | Reference | 635/14295 | Reference | Reference |
| Medium (4-5) | 704/18450 | 0.85 (0.77-0.95) | 0.89 (0.80-0.99) | 696/18321 | 0.85 (0.77-0.95) | 0.89 (0.80-0.99) |
| High (6-9) | 319/9479 | 0.77 (0.67-0.88) | 0.82 (0.71-0.94) | 309/9415 | 0.76 (0.66-0.87) | 0.81 (0.71-0.93) |
| Per unit increase | 1667/42327 | 0.94 (0.91-0.96) | 0.95 (0.92-0.98) | 1640/42031 | 0.93 (0.91-0.96) | 0.95 (0.92-0.98) |

^1^HRs (hazard ratios) and 95% CI (confidence intervals) were derived from Cox models using attained age as the time scale, adjusted for year of birth (1942-46, 1947-51, 1952-56 and 1957-62).

^2^ HRs (hazard ratios) and 95% CI (confidence intervals) were derived from Cox models using attained age as the time scale, adjusted for year of birth, body mass index, smoking habit, physical activity, total energy intake, education years, diabetes, hypertension. Reference: The category used as comparison.

MDP: Mediterranean dietary pattern.

**Table S3. The association between adherence to the Mediterranean dietary pattern and the risk of depression adjusted for history of other psychiatric disorder**

|  | Depression | | |
| --- | --- | --- | --- |
| Adherence to MDP | Case/participants, N | Minimally adjusted HR (95% CI)^1^ | Fully adjusted HR (95% CI)^2^ |
|  |  |  |  |
| Low (0-3) | 646/14,453 | Reference category | Reference category |
| Medium (4-5) | 710/18,540 | 0.86 (0.77-0.96) | 0.93 (0.83-1.03) |
| High (6-9) | 321/9,522 | 0.77 (0.67-0.88) | 0.84 (0.73-0.96) |
| Per unit increase | 1,677/42,515 | 0.94 (0.91-0.97) | 0.96 (0.93-0.99) |

^1^HRs (hazard ratios) and 95% CI (confidence intervals) were derived from Cox models using attained age as the time scale, adjusted for year of birth (1942-46, 1947-51, 1952-56 and 1957-62).

^2^ HRs (hazard ratios) and 95% CI (confidence intervals) were derived from Cox models using attained age as the time scale, adjusted for year of birth, body mass index, smoking habit, physical activity, total energy intake, education years, diabetes, hypertension and history of any other psychiatric disorder.

MDP: Mediterranean dietary pattern.

**Table S4. The association between adherence to the Mediterranean dietary pattern and the risk of depression among women without psychiatric history**

|  | Depression | | |
| --- | --- | --- | --- |
| Adherence to MDP | Case/participants, N | Minimally adjusted HR (95% CI)^2^ | Fully adjusted HR (95% CI)^3^ |
|  |  |  |  |
| Low (0-3) | 358/13,889 | Reference category | Reference category |
| Medium (4-5) | 419/17,897 | 0.91 (0.79-1.05) | 0.92 (0.80-1.06) |
| High (6-9) | 190/9,149 | 0.81 (0.68-0.97) | 0.83 (0.69-0.99) |
| Per unit increase | 967/40,935 | 0.95 (0.91-0.99) | 0.95 (0.92-0.99) |

^1^Subjects with any psychiatric disorder history before cohort entry were excluded (n=1908).

^2^HRs (hazard ratios) and 95% CI (confidence intervals) were derived from Cox models using attained age as the time scale, adjusted for year of birth (1942-46, 1947-51, 1952-56 and 1957-62).

^3^ HRs (hazard ratios) and 95% CI (confidence intervals) were derived from Cox models using attained age as the time scale, adjusted for year of birth, body mass index, smoking habit, physical activity, total energy intake, education years, diabetes and hypertension.

MDP: Mediterranean dietary pattern.

**Table S5. The association between adherence to the Mediterranean dietary pattern (based on red and processed meat) and the risk of depression**

|  | Depression | | |
| --- | --- | --- | --- |
| Adherence to MDP | cases/participants, N | Minimally adjusted HR (95% CI)^1^ | Fully adjusted HR (95% CI)^2^ |
|  |  |  |  |
| Low (0-3) | 646/14,453 | Reference category | Reference category |
| Medium (4-5) | 710/18,540 | 0.86 (0.78-0.96) | 0.90 (0.81-1.01) |
| High (6-9) | 321/9,522 | 0.77 (0.67-0.88) | 0.82 (0.71-0.94) |
| Per unit increase | 1,677/42,515 | 0.94 (0.91-0.97) | 0.95 (0.93-0.98) |

^1^HRs (hazard ratios) and 95% CI (confidence intervals) were derived from Cox models using attained age as the time scale, adjusted for year of birth (1942-46, 1947-51, 1952-56 and 1957-62).

^2^HRs (hazard ratios) and 95% CI (confidence intervals) were derived from Cox models using attained age as the time scale, adjusted for year of birth, body mass index, smoking habit, physical activity, total energy intake, education years, diabetes and hypertension.

MDP: Mediterranean dietary pattern (only included red and processed meat in the meat component).

|  | **Table S6 The consumption of different components and their correlations with the score of Mediterranean dietary pattern.** | | |  |
| --- | --- | --- | --- | --- |
| Component | | Consumption  (Median, g/d) | Spearman's r^1^ | |
|  |  |  |  |  |
| Vegetables | | 61.95 | 0.42 | |
| Fruits and nuts | | 136.66 | 0.37 | |
| Legumes | | 17.54 | 0.39 | |
| Cereals | | 182.80 | 0.24 | |
| Fish and seafood | | 22.69 | 0.41 | |
| Meat | | 84.42 | 0.04 | |
| Red and processed meat | | 76.51 | 0.01 | |
| Dairy products | | 333.73 | -0.28 | |
| M/S ratio | | 0.75 | 0.35 | |
| Alcohol | | 1.79 | 0.16 | |

^1^Spearman correlation coefﬁcients of component with score of Mediterranean dietary pattern.

M/S: Monounsaturated fat/saturated fat

**Table S7 The age specific analysis over the first 10 years and the second 10 years of follow-up**

| Adherence to MDP | Cases, N | Minimally adjusted HR (95% CI)^1^ | Fully adjusted HR (95% CI)^2^ |
| --- | --- | --- | --- |
| **The first 10-year follow-up** |  |  |  |
| **Age at follow-up <50** |  | | |
| Low (0-3) | 54 | reference category. | reference category. |
| Medium (4-5) | 73 | 1.11 (0.78-1.57) | 1.16 (0.81-1.65) |
| High (6-9) | 31 | 1.00 (0.64-1.55) | 1.07 (0.68-1.68) |
| **Age at follow-up ≥50** |  | | |
| Low (0-3) | 28 | reference category. | reference category. |
| Medium (4-5) | 40 | 0.98 (0.60-1.58) | 0.94 (0.58-1.54) |
| High (6-9) | 20 | 0.82 (0.46-1.46) | 0.77 (0.43-1.38) |
| **The second 10-year follow-up** |  |  |  |
| **Age at follow-up <50** |  | | |
| Low (0-3) | 169 | reference category. | reference category. |
| Medium (4-5) | 148 | 0.80 (0.64-1.00) | 0.87 (0.70-1.09) |
| High (6-9) | 68 | 0.86 (0.65-1.13) | 1.00 (0.75-1.33) |
| **Age at follow-up ≥50** |  | | |
| Low (0-3) | 395 | reference category. | reference category. |
| Medium (4-5) | 449 | 0.85 (0.74-0.97) | 0.88 (0.77-1.01) |
| High (6-9) | 202 | 0.72 (0.61-0.85) | 0.76 (0.64-0.91) |

^1^HRs (hazard ratios) and 95% CIs (confidence intervals) were derived from Cox models using attained age as the underlying time scale, adjusted for year of birth in 5-year intervals (1942-46, 1947-51, 1952-56 and 1957-62). Estimated the risks separately in the population over the first and the second 10 years of follow-up at age <50 and ≥50.

^2^HRs (hazard ratios) and 95% CIs (confidence intervals) were derived from Cox models using attained age as the underlying time scale, adjusted for year of birth in 5-year intervals (1942-46, 1947-51, 1952-56 and 1957-62), body mass index, smoking, physical activity, total energy intake, years of education, diabetes, and hypertension. Estimated the risks separately in the population over the first and the second 10 years of follow-up at age <50 and ≥50.

MDP: Mediterranean dietary pattern; N: number.

**SAS codes for the main Cox regression analyses**

*---------create dataset for survival analysis---------;

data m1;

set sasperm.ana1;

agein=(nsstartd-birth_dat)/365.25;

ageout=(cdate-birth_dat)/365.25;

keep lpnr event birth_cat agein ageout sum_score1 score_cat n2 cbmi physact smoker ceduc diabetes hypert;

run;

*---Minimally adjusted Cox model---;

*---Using attained age at the underlying time-scale, further adjusted for calendar year of birth---;

*---Continuous score (0-9)---;

proc phreg data=m1;

class birth_cat (ref='1942-46');

model (agein, ageout)*event(0)= sum_score1 birth_cat / risklimits rl=pl;

title 'timescale=attained age, continuous score, minimally adjusted';

run;

*---Adherence categories (low/0-3, medium/4-5 and high/6-9)---;

proc phreg data=m1;

class score_cat (ref='0-3')

birth_cat (ref='1942-46');

model (agein, ageout)*event(0)= score_cat birth_cat / risklimits rl=pl;

title 'timescale=attained age, score categories , minimally adjusted';

run;

*---Fully adjusted model---;

*---Using attained age at the underlying time-scale, further adjusted for calendar year of birth, body mass index, smoking, physical activity, total energy intake, years of education, diabetes, and hypertension ---;

*---Continuous score (0-9)---;

proc phreg data=m1;

class cbmi (ref='<25')

physact (ref='Moderate')

smoker (ref='Never')

ceduc (ref='0-10yrs')

diabetes (ref='No')

hypert (ref='No')

birth_cat (ref='1942-46');

model (agein, ageout)*event(0)= sum_score1 n2 cbmi physact smoker ceduc diabetes hypert birth_cat / risklimits rl=pl;

title 'timescale=attained age, continuous score, fully adjusted';

run;

*---Adherence categories (low/0-3, medium/4-5 and high/6-9)---;

proc phreg data=m1;

class score_cat (ref='0-3')

cbmi (ref='<25')

physact (ref='Moderate')

smoker (ref='Never')

ceduc (ref='0-10yrs')

diabetes (ref='No')

hypert (ref='No')

birth_cat (ref='1942-46');

model (agein, ageout)*event(0)= score_cat n2 cbmi physact smoker ceduc diabetes hypert birth_cat / risklimits rl=pl;

title 'timescale=attained age, score categories, fully adjusted';

run;
